# Patient costs of breast cancer endocrine therapy agents under Medicare Part D vs with generic formulations

**DOI:** 10.1186/s40064-015-0827-8

**Published:** 2015-02-03

**Authors:** Ann Butler Nattinger, Liliana E Pezzin, Emily L McGinley, John A Charlson, Tina W F Yen, Joan M Neuner

**Affiliations:** 1 Division of General Internal Medicine, Medical College of Wisconsin, 8701 Watertown Plank Road, Milwaukee, WI USA; 2 Division of Hematology-Oncology, Medical College of Wisconsin, 8701 Watertown Plank Road, Milwaukee, WI USA; 3 Division of Surgical Oncology, Medical College of Wisconsin, 8701 Watertown Plank Road, Milwaukee, WI USA; 4 Center for Patient Care and Outcomes Research, Medical College of Wisconsin, 8701 Watertown Plank Road, Milwaukee, WI 53226 USA

**Keywords:** Breast cancer, Treatment, Hormonal therapy, Cost, Medicare

## Abstract

**Purpose:**

The high expense of newer, more effective adjuvant endocrine therapy agents (aromatase inhibitors [AIs]) for postmenopausal breast cancer contributes to socioeconomic disparities in breast cancer outcomes. This study compares endocrine therapy costs for breast cancer patients during the first five years of Medicare Part D implementation, and when generic alternatives became available.

**Methods:**

The out of pocket patient costs for AIs and tamoxifen under Medicare Part D drug plans were determined for 2006–2011 from the CMS Website for the 50 US states and District of Columbia.

**Results:**

Between 2006 and 2010, the mean annual patient drug cost under Medicare Part D in the median state rose 19% for tamoxifen, 113% for anastrozole, 89% for exemestane, and 129% for letrozole, resulting in median annual out of pocket costs in 2010 of $701, $3050, $2804, and $3664 respectively. However, the 2011 availability of generic AI preparations led to median annual costs in 2011 of $804, $872, $1837, and $2217 respectively. Not included in the reported patient costs, the mean monthly drug premiums in the median state increased 58% in 2011 compared to 2007.

**Conclusions:**

The more effective AI agents became considerably more expensive during the first several years of the Medicare Part D program. Cost decreased with the introduction of generic agents, an intervention that was independent of the Part D program. It is unlikely that the Part D program ameliorated existing socioeconomic disparities in survival among breast cancer patients, but the availability of generic agents may do so.

## Introduction

Adjuvant oral endocrine therapy for breast cancer represents one of the most important advances in treatment in the past several decades. The efficacy of tamoxifen for the 75% of postmenopausal breast cancer patients with hormone receptor positive disease was initially demonstrated by the mid-1980’s, and dozens of trials have shown that such women randomized to 5 years of tamoxifen therapy have a 47% reduction in breast cancer recurrence and 26% reduction in mortality at 10 years compared to placebo (Early Breast Cancer Trialists’ Collaborative Group 1998; Early Breast Cancer Trialists’ Collaborative Group 2005). More recently, trials have demonstrated that aromatase inhibitor (AI) agents further reduce breast cancer recurrence rates by 30-50% compared to tamoxifen alone among postmenopausal women with hormone receptor positive disease (Dowsett et al. 2010). Since early 2005, the American Society of Clinical Oncology has recommended that all such women be offered AI therapy either alone or in sequential combination with tamoxifen (Winer et al. 2005); other influential groups have made similar recommendations (Carlson et al. 2006).

While the AI agents are more effective than tamoxifen in preventing breast cancer recurrence, these drugs have historically been substantially more costly. This cost differential has led to marked socioeconomic disparities in their use. For example, the use of AI agents (compared to tamoxifen) is more likely among breast cancer survivors who are wealthier, who have pharmaceutical insurance coverage, and who have better social support, despite adjustment for a variety of demographic factors (Yen et al. 2011). Additionally, breast cancer survivors taking AI agents are more likely to experience financial difficulty than those taking tamoxifen despite controlling for household income and drug insurance coverage (Pezzin et al. 2009).

Until 2006, the Medicare program did not provide coverage for oral, self-administered pharmaceutical agents. Effective in 2006, the Medicare Prescription Drug, Improvement and Modernization Act added a prescription drug benefit for enrollees who opted to participate in its “Part D” voluntary program providing coverage for such pharmaceutical agents (Kaiser and Foundation 2010). However, Medicare coverage of prescription drugs under Part D relies on private plans which are allowed wide discretion when setting plan features and prices. The resulting multitude of plans raises concern about variation in drug coverage and cost.

This paper documents variation in the costs to breast cancer patients of alternative Part D drug plans and of the relevant oral endocrine agents during the first several years of the Part D program, as well as the extent to which these costs changed during the first full year for which generic formulations were available. Adjuvant endocrine therapy for breast cancer is a useful area for study as there are only a limited number of pertinent oral pharmaceutical agents. In addition, the three newer, more expensive, and more effective AI agents (anastrozole, exemestane, and letrozole) can be contrasted to tamoxifen, the older, less expensive and less effective agent.

## Methods

### Source of data

We assembled information on the Part D plans available to Medicare beneficiaries in each state and the District of Columbia by manually querying the Centers for Medicare and Medicaid Services (CMS) website used by Medicare beneficiaries to find and compare the Part D plans available in their areas (Medicare Plan Finder). For each plan in each state, information was collected on deductible amounts, monthly drug premiums and the specific drug costs for each of the four endocrine agents of interest (anastrozole, exemestane, letrozole, and tamoxifen). Drug specific costs collected included the drug cost after the deductible was met, the cost in the coverage gap (also known as “doughnut hole”, and reached after about $2500-3100 [exact amount changes annually] of out of pocket drug costs in a calendar year), and the cost under catastrophic coverage (which is reached after expending about $4500 [exact amount changes annually] of out-of-pocket drug costs in a calendar year). The annual drug cost under each plan was also obtained from the website and represents the aggregate cost to a beneficiary who took only that drug at recommended dosing for the entire year, including deductible and drug-specific costs, but not the plan premiums. The project was approved by the Medical College of Wisconsin/Froedtert Hospital Institutional Review Board #5.

### Data analysis

While the Medicare Part D program initiated coverage in 2006, the program provided only partial coverage for many patients that year; 2007 was the first full year of coverage for most beneficiaries. In this paper, we focus on contrasting costs faced by breast cancer patients in 2006 or 2007 (first full year of Part D implementation) to 2010 (fifth year of Part D implementation) and 2011 (first full year of availability of generic AI agents on CMS Website). Specifically, the state-specific mean monthly drug premium costs were computed for plans in each state. We report the median and range of the state-specific mean premium costs for each study year. Similarly, for the drug-specific costs, the mean costs were computed for each state, and we report the median and range of these state means.

## Results

### Costs of breast cancer endocrine agents

The drug-specific costs of the adjuvant endocrine agents most commonly used for early stage breast cancer are presented for 2007, 2010, and 2011 (Tables 1 and 2). Four drugs are of interest, specifically, the three FDA-approved AI agents (anastrozole, exemestane, and letrozole) and tamoxifen. Only tamoxifen was available in generic formulation on the CMS website during 2007–10, but generic agents were available for each of the AI agents during 2011. All states had one or more plans providing coverage for each of these medications; typically, all plans in a state provided coverage for each medication.

**Table 1:** **Trajectory of costs under Medicare Part D for breast cancer adjuvant endocrine agents prior to generic availability**

**Medication**	**Monthly cost in gap**			**Monthly cost in catastrophic**					
	**Median of state mean costs**			**Median of state mean costs**			**Median of state mean costs**		
	**$, (Range of mean costs)**			**$, (Range of mean costs)**			**$, (Range of mean costs)**		
	**2007**	**2010**	**Change (%)**	**2007**	**2010**	**Change (%)**	**2007**	**2010**	**Change (%)**
Arimidex	39.64 (38.37-48.19)	62.83 (60.00-74.75)	+58	258.64 (247.44-263.47)	392.84 (375.66-418.58)	+52	13.18 (12.68-13.32)	19.65 (19.03-20.93)	+49
Aromasin	42.57 (40.72-50.94)	86.50 (81.51-101.08)	+103	267.56 (255.21-273.62)	353.98 (351.86-377.07)	+32	13.66 (13.07-13.79)	17.70 (17.59-18.85)	+30
Femara	41.92 (39.38-51.11)	89.03 (80.95-102.77)	+112	277.65 (249.14-282.54)	433.42 (430.46-461.42)	+56	14.16 (12.96-14.34)	21.67 (21.55-23.07)	+53
Tamoxifen	6.19 (5.95-9.22)	5.98 (5.66-6.75)	−3	21.32 (20.36-25.42)	15.52 (15.03-18.90)	−27	2.26 (2.20-2.70)	2.60 (2.58-2.75)	+15

**Table 2:** **Trajectory of costs under Medicare Part D for breast cancer adjuvant endocrine agents before and after availability of generic alternatives**

**Medication**	**Monthly cost in gap**			**Monthly cost in catastrophic**					
	**Median of state mean costs**			**Median of state mean costs**			**Median of state mean costs**		
	**$, (Range of mean costs)**			**$, (Range of mean costs)**			**$, (Range of mean costs)**		
	**2007**	**2011** ^**a**^	**Change (%)**	**2007**	**2011** ^**a**^	**Change (%)**	**2007**	**2011** ^**a**^	**Change (%)**
Arimidex/Anastrozole	39.64 (38.37-48.19)	9.57 (8.00-11.95)	−76	258.64 (247.44-263.47)	38.94 (31.80-78.46)	−85	13.18 (12.68-13.32)	3.72 (3.39-6.19)	−72
Aromasin/Exemestane	42.57 (40.72-50.94)	49.50 (42.34-118.68)	+16	267.56 (255.21-273.62)	244.51 (228.61-270.95)	−9	13.66 (13.07-13.79)	14.41 (13.51-71.41)	+6
Femara/Letrozole	41.92 (39.38-51.11)	69.31 (59.69-85.17)	+65	277.65 (249.14-282.54)	225.13 (209.10-252.23)	−19	14.16 (12.96-14.34)	38.53 (33.70-57.15)	+172
Tamoxifen	6.19 (5.95-9.22)	5.54 (5.12-6.89)	−11	21.32 (20.36-25.42)	13.17 (12.44-21.11)	−38	2.26 (2.20-2.70)	2.73 (2.50-3.47)	+21

^a^First year generic formulation was available to Medicare Part D beneficiaries; generic cost provided.

For each plan covering a medication, three costs were consistently provided for each drug. These were the monthly cost after the deductible, the monthly cost in the gap (or “doughnut hole”), and the monthly cost once catastrophic expense levels were reached. In 2007, nine states offered at least one “no-cost” plan (that is, after payment of the deductible, drug offered at no cost until reaching the doughnut hole) for the three AI agents. In 2010 and 2011, no plans in any state offered a no-cost plan for AI coverage. In contrast, for tamoxifen, at least one plan in each state offered a no-cost plan in each study year.

In 2007, the median monthly cost after deductible was about $40 for each of the three AI agents (Tables 1 and 2). By 2010, this cost had increased by 58% for anastrozole and by over 100% for exemestane and letrozole (Table 1). However in 2011, the first full year of generic availability of the AI’s, the monthly cost after deductible for anastrazole fell to 76% less than the 2007 cost. The monthly cost for exemestane and letrozole in 2011 moderated compared to 2010, but did remain higher than the 2007 costs (Table 2). The monthly cost for tamoxifen fell by less than $1 in both 2010 and 2011.

The cost in the gap of the three aromatase inhibitors increased from 32% to 56% between 2007 and 2010, but these costs fell between 9 and 85% between 2007 and 2011. In contrast the cost in the gap of tamoxifen, already less than one-tenth of the aromatase inhibitor cost in 2007, fell by 27% in 2010 and by 38% in 2011. Costs for all medications were much more moderate in the catastrophic phase. These costs in 2011 compared to 2007 declined for anastrazole, remained about the same for exemestane, and rose for letrozole and tamoxifen.

### Annual drug costs

The annual drug costs represent the aggregate cost to the patient of taking only a given drug at recommended dosing for an entire year, including deductible and drug-specific costs, but not the plan premiums. Annual drug costs were not available for 2007; Table 3, therefore, reports the annual drug costs for the three AI agents and for tamoxifen in 2006, 2010, and 2011. During the first five years of the Part D drug plan, the median annual drug cost to the patient for tamoxifen (generic) increased 19% to $701.18 while the median annual drug cost for anastrozole, exemestane, and letrozole (brand; no generics provided) rose 113%, 89%, and 129% (to $3050, $2804, and $3664) respectively. In 2011, the first year of generic AI availability on the CMS Website, the annual cost to the patient of anastrozole fell 39% from the 2006 level and 71% from the 2010 level. The costs for exemestane and letrozole declined more modestly from the 2010 levels, to costs 24% and 39% above the 2006 levels respectively. In 2006, the ratio of annual cost to the patient for AI compared to tamoxifen was 2.4, 2.5, and 2.7 for anastrozole, exemestane, and letrozole respectively. In 2010, these ratios were 4.4, 4.0, and 5.2, but in 2011 these ratios fell to 1.1, 2.3, and 2.8 respectively.

**Table 3:** **Annual drug costs for breast cancer adjuvant endocrine agents under medicare Part D and after availability of generic alternatives**

	**2006 annual costs** ^**a**^	**2010 annual costs** ^**a**^	**2006 to 2010**	**2011 annual costs** ^**a,b**^	**2006 to 2011**	**2010 to 2011**
**Drug**	**Median of state mean costs**	**Median of state mean costs**	**Change in median cost (%)**	**Median of state mean costs**	**Change in median cost (%)**	**Change in median cost (%)**
				**$, (Range of mean costs)**		
	**$, (Range of mean costs)**	**$, (Range of mean costs)**				
Arimidex/Anastrozole	1432.96 (1345.26-1547.73)	3050.37 (2943.92-3365.10)	+113	872.20 (731.51-994.31)	−39	−71
Aromasin/Exemestane	1482.73 (1401.17-1596.30)	2804.33 (2674.05-3141.44)	+89	1836.93 (1645.53-2418.20)	+24	−34
Letrozole/Femara	1598.18 (1463.09-1681.52)	3663.61 (3535.16-4006.99)	+129	2217.49 (2083.11-2375.56)	+39	−39
Tamoxifen	588.18 (520.13-648.77)	701.18 (558.66-780.02)	+19	804.03 (660.68-881.69)	+37	+15

^a^Range and median of mean annual drug costs of Part D plans across 50 states and the District of Columbia. Annual drug cost values represent the aggregate cost to the patient of taking only that drug at recommended dosing for an entire year, including deductible and drug-specific costs, but not the plan premiums. These values were provided directly from the CMS website, and obtained by entering the relevant drug as the only drug to be taken for the year.

^b^First full year in which generic aromatase inhibitor agents were made available to Medicare Part D beneficiaries; generic cost provided.

### Plan premium costs

In 2007, the number of plans offered on the CMS Website per state ranged from 43 to 64 (median = 51) plans (Table 4). The number of plans fell to a median of 41 by 2010 and to 33 by 2011, a 35% decline from 2007 (Table 4). The monthly Part D plan premium cost is not included in the annual out of pocket drug costs shown in Table 3. The monthly premium costs increased from about $36 in 2007 to $47 in 2010 and $57 in 2011. Therefore, in addition to the annual drug costs, patients were paying a median of about $684 annually by 2011, up from $432 in 2007.

**Table 4:** **Medicare Part D plan characteristics over time**

	**2007**	**2010**	**2011** ^**a**^	**% Change in median, 2007 to 2011**
Number of Plans in Each State (Median; Range)	51 (43–64)	41 (34–49)	33 (28–38)	−35.3
Mean Monthly Drug Premium in Each State (Median $, Range)	36.09 (31.72-40.69)	46.72 (35.12-53.01)	57.08 (44.93-62.97)	+58.2

^a^First full year when generic alternatives to brand name aromatase inhibitors were made available to Medicare Part D beneficiaries.

## Discussion

This study documents substantial increases of 89% to 129% in the annual cost to Medicare beneficiaries with breast cancer of the more effective aromatase inhibitor agents over the first five years of the Medicare Part D drug program. However after the 2011 introduction of generic AI agents, a private sector event unrelated to Part D, beneficiaries’ costs were much more moderate. For example, the median out of pocket cost to beneficiaries of anastrozole was $1433 annually in 2006, rose to $2804 in 2010, and then declined to $872 in 2011. The relative cost of the three AI agents compared to the less effective tamoxifen was 4.0 to 5.2 times greater in 2010, but fell to 1.1 to 2.8 times greater in 2011. The increase in AI costs to beneficiaries from 2006 to 2010 was attributable to increases in the monthly cost after the deductible, as well as the monthly cost in the gap and in the catastrophic phases. The very substantial cost decreases that accompanied the 2011 introduction of generic AI’s were largely attributable to the monthly cost after deductible, with lesser declines in the cost in the gap for exemestane and letrozole. Not included in the annual out of pocket drug costs, median plan premiums rose 58% in 2011 compared to 2007.

Plan benefit design represents a critical public policy tool for improving access and adherence to needed treatments. Evidence suggests that increased prescription drug cost sharing, whether in the form of co-payments, tiering, benefit caps or deductibles, is associated with lower rates of drug treatment, worse adherence among existing users and more frequent discontinuation of therapy (Goldman et al. 2004; Soumerai et al. 2006; Goldman et al. 2007; Zhang et al. 2009; Li et al. 2012). It has been estimated, for example, that for each 10% increase in cost sharing, prescription drug spending decreases by 2-6% depending on class of drug and patient’s circumstances (Goldman et al. 2007). Assuming these estimates hold for breast cancer patients, the plan and drug cost increases observed over the initial 5-year period of Part D implementation may have led to a substantial decline in use and adherence to the more effective hormonal treatments. Although the guidelines for adjuvant hormonal therapy in post-menopausal women with breast cancer permit use of tamoxifen as an endocrine agent, the recommendation is that an AI be offered for all or at least half of the minimum 5-year duration of adjuvant endocrine therapy (Winer et al. 2005; Burstein et al. 2010). With recent recommendation for 10 years of endocrine therapy for many women, the cost implications become even more important (Burstein et al. 2014). In the case of breast cancer, early discontinuation of adjuvant endocrine therapy is associated with a greater risk of recurrence and/or death (Hershman et al. 2010).

Our findings are consistent with reports suggesting that many of the largest prescription drug plans substantially raised prices since the drug benefit was added to Medicare (Kaiser and Foundation 2010; Hadley et al. 2009). These results further suggest that, at least with respect to breast cancer, drug cost increases were not universal across the range of possible oral endocrine agents but affected the more effective AI drugs to a greater degree. Attempts to streamline the Part D program in response to public criticism about the difficulty in finding the “best” Medicare prescription drug plan, including eliminating duplicative plans, did result in lower overall number of plans offered in each market as evidenced by our 2010–11 results, but may have contributed to increased costs to beneficiaries.

An important goal of the Medicare Part D program was to reduce medication and socioeconomic-related disparities in health outcomes (Kaiser and Foundation 2010). An important provision of the later enacted Patient Protection and Affordable Care Act was to establish additional help to low income beneficiaries in the form of the Low Income Subsidy (LIS), a benefit that provides full or partial subsidies of premiums and reductions in cost-sharing for Part D. The challenges posed by the Medicare Part D program enrollment and plan choice, however, appear to be even greater among this group (Davidoff et al. 2010). Although Medicare beneficiaries with full Medicaid benefits are automatically enrolled in Part D, an estimated 2.3 million beneficiaries eligible for the LIS have not applied for the subsidy (Enrollment Information 2009). Evidence further suggests that those receiving the LIS benefit experience greater difficulty navigating program information and making decisions about plans: compared to other Part D beneficiaries, LIS recipients are less likely to understand plan features, including premiums and deductibles, or their rights under Part D, including the right to enroll in a different plan (Neuman et al. 2007; Hsu et al. 2008).

Previous studies have shown that socioeconomic status, particularly household income, is related to use of AI agents. Wealthier women and those with more extensive insurance coverage are more likely to receive AI therapy (Yen et al. 2011). Residence in a high poverty area is a risk factor for failure to receive guideline concordant hormonal therapy (Wu et al. 2012). High out-of-pocket cost has been found to be strongly associated with non-adherence with hormonal therapy agents for breast cancer (Riley et al. 2011; Hershman et al. 2014). These findings suggest that the cost of AI agents contributes to the ongoing socioeconomic disparities in survival among breast cancer patients (Bigby and Holmes 2005). Against the backdrop of low uptake of the LIS benefit and lack of understanding about the Part D program among low-income beneficiaries, our results raise questions about whether the Part D program has led to any amelioration of existing socioeconomic disparities in breast cancer outcomes. It is likely that the introduction of generic agents was more effective in mitigating such disparities in breast cancer outcomes.

Our data were gathered from the publicly available CMS web site used by beneficiaries to compare different plans available to them. As such, the data represent the range of choices available to beneficiaries, but not the actual choices made by those beneficiaries. The distribution of costs in the plans actually selected by beneficiaries would be expected to differ somewhat from the distribution of costs presented herein, as plans are not equally selected by beneficiaries. In fact, the Kaiser Foundation conducted an analysis of plans that CMS expected to be available for 2010, with weighting based on expected enrollment (Hadley et al. 2009). The projected weighted monthly drug plan premium based on this analysis was $38.94, which is lower than the median of the state premiums, which we report to be $46.72 (Table 4). This implies that Part D participants are choosing plans with lower premiums than the mean of the available plans. Supporting this contention, Abaluck and Gruber demonstrated that elderly persons systematically make inefficient drug plan choices and tend to place more weight on plan premiums than on expected out of pocket costs (Abaluck and Gruber 2011). Therefore the median annual drug costs reported in this paper may underestimate the actual median annual drug costs for the population of Medicare beneficiaries using Part D programs to purchase endocrine agents for breast cancer.

A limitation of our study is that the CMS web site may have some inaccuracies. Updates are made to the web site throughout the calendar year, and these updates would not necessarily have been captured by us, as we queried the website early in each calendar year. However, because most Part D participants select their plans in November or December for the following calendar year, any updates made during the calendar year are unlikely to affect the plan choices made by most participants for that year. Therefore, it is likely that the information we gathered was the same information used by the majority of Medicare beneficiaries to make a plan choice for that calendar year.

Early evaluations of the Medicare Part D program have been generally positive. For example, there is evidence that cost-related non-adherence fell somewhat in 2006 compared to 2004–5, (Madden et al. 2008) although this decrease was observed only among beneficiaries in good to excellent health, and not beneficiaries in fair to poor health. The implementation of Part D coverage was associated with a reduction in nondrug medical spending in 2006–07, compared to 2004–05, for beneficiaries with limited prior drug coverage (McWilliams et al. 2011). Satisfaction with the Part D program was high in 2007 (Keenan 2007). However, we show that the situation in 2010 was quite a bit different than in 2006, at least for breast cancer patients. The substantive changes in beneficiary costs provide justification for continued evaluation of the effectiveness of the program with regard to multiple constructs.

In conclusion, the costs under the Medicare Part D program of aromatase inhibitors, the most effective breast cancer adjuvant endocrine agents, rose dramatically between 2006 and 2010, and then fell with the availability of generic AI agents in 2011. These results raise concern about the degree to which the Medicare Part D plan will achieve its goals of improving accessibility to life-saving pharmaceutical agents, and decreasing socioeconomic disparities in outcomes. Rather, the availability of generic preparations may be more likely to achieve these aims.

### Ethical standards

All studies comply with the current laws of the United States in which they were performed.

## References

[CR1] Abaluck J, Gruber J (2011). Choice inconsistencies among the elderly: evidence from plan choice in the Medicare Part D program. Am Econ Rev.

[CR2] Bigby J, Holmes M (2005). Disparities across the breast cancer continuum. Cancer Causes Control.

[CR3] Burstein H, Prestrud AA, Seidenfeld J, Anderson H, Buchholz TA, Davidson NE, Gelmon KE, Giordano S, Hudis C, Malin J, Mamounas E, Rouden D, Solky A, Sowers M, Somerfield MR, Griggs JJ (2010). American society of clinical oncology clinical practice guideline: update on adjuvant endocrine therapy for women with hormone receptor-positive breast cancer. J Clin Oncol.

[CR4] Burstein HJ, Temin S, Anderson H, Buchholz TA, Davidson NE, Gelmon KE, Giordano S, Hudis C, Rowden D, Solky A, Stearns V, Winer E, Griggs JJ (2014). Adjuvant Endocrine Therapy for Women with Hormone Receptor-Positive Breast Cancer: American Society of Clinical Oncology Clinical Practice Guideline Focused Update. J Clin Oncol.

[CR5] Carlson RW, Hudis CA, Pritchard KI (2006). National comprehensive cancer network breast cancer clinical practice guidelines in O, American society of clinical oncology technology assessment on the use of Aromatase I, St Gallen international expert consensus on the primary therapy of early breast C. Adjuvant endocrine therapy in hormone receptor-positive postmenopausal breast cancer: evolution of NCCN, ASCO, and St Gallen recommendations. J Natl Compr Canc Netw.

[CR6] Davidoff AJ, Stuart B, Shaffer T, Shoemaker JS, Kim M, Zacker C (2010). Lessons learned: who didn’t enroll in Medicare drug coverage in 2006, and why?. Health Aff.

[CR7] Dowsett M, Cuzick J, Ingle J, Coates A, Forbes J, Bliss J, Buyse M, Baum M, Buzdar A, Colleoni M, Coombes C, Snowdon C, Gnant M, Jakesz R, Kaufmann M, Boccardo F, Godwin J, Davies C, Peto R (2010). Meta-analysis of breast cancer outcomes in adjuvant trials of aromatase inhibitors versus tamoxifen. J Clin Oncol.

[CR8] Early Breast Cancer Trialists’ Collaborative Group (1998). Tamoxifen for early breast cancer: an overview of the randomised trials. Lancet.

[CR9] Early Breast Cancer Trialists’ Collaborative Group (2005). Effects of radiotherapy and of differences in the extent of surgery for early breast cancer on local recurrence and 15-year survival: an overview of the randomised trials. Lancet.

[CR10] Enrollment Information. 2009. (Accessed February 1, 2009, at http://www.cms.hhs.gov/PrescriptionDrugCovGenIn)

[CR11] Goldman D, Joyce G, Escarce J, Pace JE, Solomon MD, Laouri M, Landsman PM, Teutsch SM (2004). Pharmacy benefits and the use of drugs by the chronically ill. JAMA.

[CR12] Goldman D, Joyce G, Zheng Y (2007). Prescription drug cost sharing: associations with medication and medical utilization and spending and health. JAMA.

[CR13] Hadley J, Cubanski J, Hargarave E, Summer L, Neuman T (2009) Part D plan availability in 2010 and key changes since 2006 (Publication #7986)

[CR14] Hershman DL, Shao T, Kushi LH, Shao T, Buono D, Kershenbaum A, Tsai W, Fehrenbacher L, Gomez SL, Miles S, Neugut AI (2010). Early discontinuation and nonadherence to adjuvant hormonal therapy in a cohort of 8,769 early-stage breast cancer patients. J Clin Oncol.

[CR15] Hershman DL, Tsui J, Meyer J, Glied S, Hillyer GC, Wright JD, and Neugut AI (2014) The Change From Brand-Name to Generic Aromatase Inhibitors and Hormone Therapy Adherence for Early-Stage Breast Cancer. J Nat Cancer Inst 106 (11) Available from: URL: http://dx.doi.org/10.1093/jnci/dju319 10.1093/jnci/dju319 PMC4271034 25349080

[CR16] Hsu J, Fung V, Price M, Huang J, Brand R, Hui R, Fireman B, Newhouse JP (2008). Medicare beneficiaries' knowledge of Part D prescription drug program benefits and responses to drug costs. JAMA.

[CR17] Kaiser Family Foundation (2010) Medicare: A Primer. Report #615-03

[CR18] Keenan T (2007) Prescription Drugs and Medicare Part D: A Report on Access, Satisfaction, and Cost. AARP, Knowledge Management.

[CR19] Li P, McElligott S, Bergquist H, Schwartz JS, Doshi JA (2012). Effect of the Medicare Part D coverage gap on medication use among patients with hypertension and hyperlipidemia. Ann Intern Med.

[CR20] Madden J, Graves A, Zhang F, Adams AS, Briesacher BA, Ross-Degnan D, Gurwitz JH, Adler GS, Somerai SB (2008). Cost-related medication nonadherence and spending on basic needs following implementation of Medicare Part D. JAMA.

[CR21] McWilliams J, Zaslavsky A, Huskamp H (2011). Implementation of Medicare Part D and nondrug medical spending for elderly adults with limited prior drug coverage. JAMA.

[CR22] Medicare Plan Finder. (Accessed at www.medicare.gov/find-a-plan/questions/home.aspx)

[CR23] Neuman P, Strollo M, Guterman S, Rogers WH, Li A, Rodday AM, Gelb Safran D (2007). Medicare prescription drug benefit progress report: findings from a 2006 national survey of seniors. Health Aff.

[CR24] Pezzin LE, O’Niel M, Nattinger AB (2009). The economic consequences of alternative breast cancer adjuvant hormonal treatments. J Gen Intern Med.

[CR25] Riley GF, Warren JL, Harlan LC, Blackwell SA (2011). Endocrine therapy use among elderly hormone receptor-positive breast cancer patients enrolled in Medicare Part D. Medicare Medicaid Res Rev.

[CR26] Soumerai S, Pierre-Jacques M, Zhang F, Ross-Degnan D, Adams AS, Gurwitz J, Adler G, Gelb Safran D (2006). Cost-related medication nonadherence among elderly and disabled Medicare beneficiaries: a national survey 1 year before the Medicare drug benefit. Arch Intern Med.

[CR27] Winer EP, Hudis C, Burstein HJ, Wolff AC, Pritchard KI, Ingle JN, Chlebowski RT, Gelber R, Edge SB, Gralow J, Cobleigh MA, Mamounas EP, Goldstein LJ, Whelan TJ, Powles TJ, Bryant J, Perkins C, Perotti J, Braun S, Langer AS, Browman GP, Somerfield MR (2005). American society of clinical oncology technology assessment on the use of aromatase inhibitors as adjuvant therapy for postmenopausal women with hormone receptor-positive breast cancer: status report 2004. J Clin Oncol.

[CR28] Wu X-C, Lund MJ, Kimmick GG, Richardson LC, Sabatino SA, Chen VW, Fleming ST, Trentham-Diez A, Lipscomb J (2011). Influence of race, insurance, socioeconomic status, and hospital type on receipt of guideline-concordant adjuvant systemic therapy for locoregional breast cancers. J Clin Oncol.

[CR29] Yen TW, Czypinski LK, Sparapani RA, Guo C, Laud PW, Pezzin LE, Nattinger AB (2011). Socioeconomic factors associated with adjuvant hormone therapy use in older breast cancer survivors. Cancer.

[CR30] Zhang Y, Donohue JM, Newhouse JP, Lave JR (2009). The effects of the coverage gap on drug spending: a closer look at Medicare Part D. Health Aff.

