# Erratum to: Patient costs of breast cancer endocrine therapy agents under Medicare Part D vs with generic formulations

**DOI:** 10.1186/s40064-015-1233-y

**Published:** 2015-09-21

**Authors:** Ann Butler Nattinger, Liliana E Pezzin, Emily L McGinley, John A Charlson, Tina W F Yen, Joan M Neuner

**Affiliations:** Division of General Internal Medicine, Medical College of Wisconsin, 8701 Watertown Plank Road, Milwaukee, WI USA; Division of Hematology-Oncology, Medical College of Wisconsin, 8701 Watertown Plank Road, Milwaukee, WI USA; Division of Surgical Oncology, Medical College of Wisconsin, 8701 Watertown Plank Road, Milwaukee, WI USA; Center for Patient Care and Outcomes Research, Medical College of Wisconsin, 8701 Watertown Plank Road, Milwaukee, WI 53226 USA

## Erratum to: SpringerPlus (2015) 4:54 DOI 10.1186/s40064-015-0827-8

It has come to our attention that during production of the original article, some of the column headings were missing from Tables 1 and 2. The corrected Tables 1 and 2 can be found below. The publisher apologises for any inconvenience caused.

**Table 1:** Trajectory of costs under Medicare Part D for breast cancer adjuvant endocrine agents prior to generic availability

Medication	Monthly cost after deductible			Monthly cost in gap			Monthly cost in catastrophic		
	Median of state mean costs			Median of state mean costs			Median of state mean costs		
	$, (Range of mean costs)			$, (Range of mean costs)			$, (Range of mean costs)		
	2007	2010	Change (%)	2007	2010	Change (%)	2007	2010	Change (%)
Arimidex	39.64 (38.37–48.19)	62.83 (60.00–74.75)	+58	258.64 (247.44–263.47)	392.84 (375.66–418.58)	+52	13.18 (12.68–13.32)	19.65 (19.03–20.93)	+49
Aromasin	42.57 (40.72–50.94)	86.50 (81.51–101.08)	+103	267.56 (255.21–273.62)	353.98 (351.86–377.07)	+32	13.66 (13.07–13.79)	17.70 (17.59–18.85)	+30
Femara	41.92 (39.38–51.11)	89.03 (80.95–102.77)	+112	277.65 (249.14–282.54)	433.42 (430.46–461.42)	+56	14.16 (12.96–14.34)	21.67 (21.55–23.07)	+53
Tamoxifen	6.19 (5.95–9.22)	5.98 (5.66–6.75)	−3	21.32 (20.36–25.42)	15.52 (15.03–18.90)	−27	2.26 (2.20–2.70)	2.60 (2.58–2.75)	+15

**Table 2:** Trajectory of costs under Medicare Part D for breast cancer adjuvant endocrine agents before and after availability of generic alternatives

Medication	Monthly cost after deductible			Monthly cost in gap			Monthly cost in catastrophic		
	Median of state mean costs			Median of state mean costs			Median of state mean costs		
	$, (Range of mean costs)			$, (Range of mean costs)			$, (Range of mean costs)		
	2007	2011^a^	Change (%)	2007	2011^a^	Change (%)	2007	2011^a^	Change (%)
Arimidex/Anastrozole	39.64 (38.37–48.19)	9.57 (8.00–11.95)	−76	258.64 (247.44–263.47)	38.94 (31.80–78.46)	−85	13.18 (12.68–13.32)	3.72 (3.39–6.19)	−72
Aromasin/Exemestane	42.57 (40.72–50.94)	49.50 (42.34–118.68)	+16	267.56 (255.21–273.62)	244.51 (228.61–270.95)	−9	13.66 (13.07–13.79)	14.41 (13.51–71.41)	+6
Femara/Letrozole	41.92 (39.38–51.11)	69.31 (59.69–85.17)	+65	277.65 (249.14–282.54)	225.13 (209.10–252.23)	−19	14.16 (12.96–14.34)	38.53 (33.70–57.15)	+172
Tamoxifen	6.19 (5.95–9.22)	5.54 (5.12–6.89)	−11	21.32 (20.36–25.42)	13.17 (12.44–21.11)	−38	2.26 (2.20–2.70)	2.73 (2.50–3.47)	+21

^a^First year generic formulation was available to Medicare Part D beneficiaries; generic cost provided

